# Transcription Factor STAT3 as a Novel Molecular Target for Cancer Prevention

**DOI:** 10.3390/cancers6020926

**Published:** 2014-04-16

**Authors:** Ailian Xiong, Zhengduo Yang, Yicheng Shen, Jia Zhou, Qiang Shen

**Affiliations:** 1 Department of Clinical Cancer Prevention, The University of Texas MD Anderson Cancer Center, Houston, TX 77030, USA; E-Mails: AXiong@mdanderson.org (A.X.); zhengduoyang@gmail.com (Z.Y.); 2 College of Natural Sciences, The University of Texas at Austin, Austin, TX 78712, USA; E-Mail: eys13094@yahoo.com; 3 Chemical Biology Program, Department of Pharmacology and Toxicology, University of Texas Medical Branch, Galveston, TX 77555, USA; E-Mail: jizhou@utmb.edu

**Keywords:** transcription factors, STAT3, cancer prevention, STAT3 inhibitors

## Abstract

Signal Transducers and Activators of Transcription (STATs) are a family of transcription factors that regulate cell proliferation, differentiation, apoptosis, immune and inflammatory responses, and angiogenesis. Cumulative evidence has established that STAT3 has a critical role in the development of multiple cancer types. Because it is constitutively activated during disease progression and metastasis in a variety of cancers, STAT3 has promise as a drug target for cancer therapeutics. Recently, STAT3 was found to have an important role in maintaining cancer stem cells *in vitro* and in mouse tumor models, suggesting STAT3 is integrally involved in tumor initiation, progression and maintenance. STAT3 has been traditionally considered as nontargetable or undruggable, and the lag in developing effective STAT3 inhibitors contributes to the current lack of FDA-approved STAT3 inhibitors. Recent advances in cancer biology and drug discovery efforts have shed light on targeting STAT3 globally and/or specifically for cancer therapy. In this review, we summarize current literature and discuss the potential importance of STAT3 as a novel target for cancer prevention and of STAT3 inhibitors as effective chemopreventive agents.

## 1. Introduction of STAT Family of Transcription Factors and STAT3

**Figure 1:**
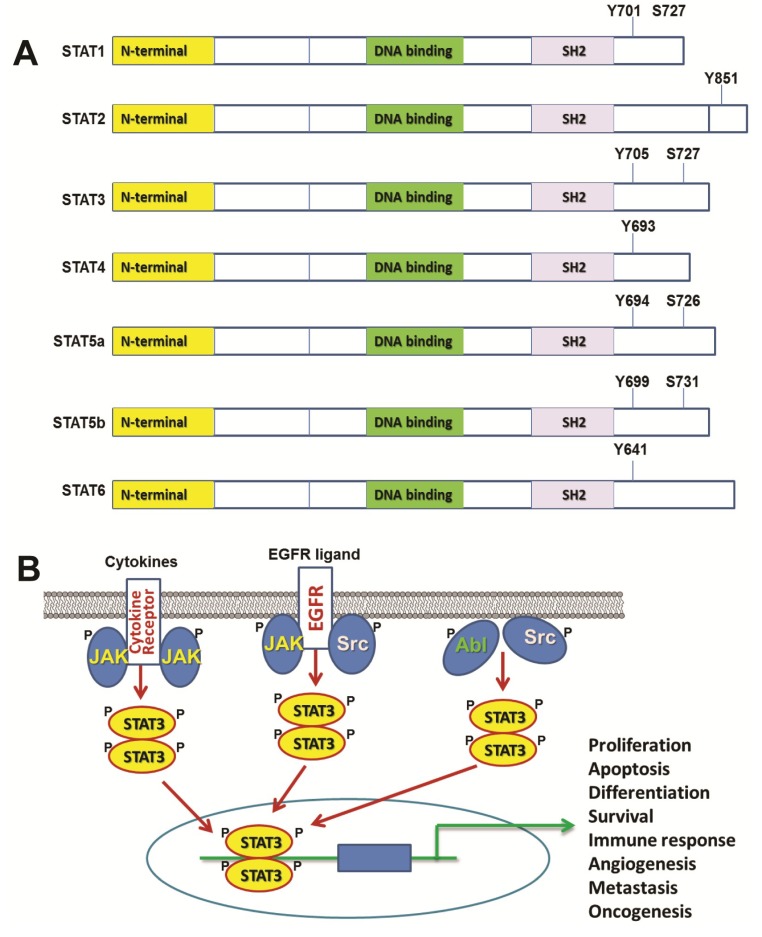
STAT family of transcription factors and STAT3 signaling pathway. (**A**) Structure and function domains for STAT1-6. (**B**) STAT3 signaling and its activation signals from extracellular and intracellular routes.

STATs, a family of transcription factors first identified in 1994 [1], play a fundamental role in the regulation of growth, survival and differentiation of various cells. Seven STAT proteins have been identified for mammalian cells: STAT1, -2, -3, -4, -5a, 5b, and -6 [2]. All STAT proteins contain a src-homology 2 (SH2) domain for binding phosphotyrosine, which is essential for activation of STAT proteins (Figure 1A). STAT transcription factors are activated by Janus kinases (JAKs). The JAK-STAT signaling pathway is frequently deregulated in primary tumors, causing increased angiogenesis, enhanced survival and immunosuppression. JAKs are cytoplasmic tyrosine kinases that are inactive in unstimulated cells. Once ligands, such as cytokines and growth factors, bind to specific receptors such as interferons IL-5 and IL-6, and epidermal growth factor receptor (EGFR) [3,4], these receptors dimerize to form a dimer complex, and recruit JAKs. The aggregation of JAKs leads to self-activation by either auto- or trans-phosphorylation. Consequently, the activated JAKs phosphorylate tyrosine residues on the cytoplasmic domain of the receptors. The phosphotyrosine on the receptor will serve as a dock for the SH2 domain of STAT proteins and recruit STAT proteins to close proximity of the JAKs. Subsequently, the STAT proteins are phosphorylated at specific tyrosine residues in the C-terminal domain and activated. Upon activation, STAT proteins form homo- or hetero-dimers via the SH2 domain and the C-terminally localized phosphotyrosine-containing domain on the partnering STAT protein. Then, the STAT dimers translocate into the nucleus and bind to specific sequences on the promoters of target genes to activate gene transcriptions [5].

Among all STAT proteins, STAT3 plays a central role in development and carcinogenesis, since it critically regulates the transcription of multiple key genes involved in cell proliferation, differentiation, apoptosis, angiogenesis, immune responses and metastasis (Figure 1B). The STAT3 gene is located in chromosome 17q21.31 [6,7]. STAT3, like other STAT family proteins, contains a dimerization domain at the N-terminus, a coiled-coil domain for protein-protein interactions, a central DNA binding domain, an SH2 domain for the recruitment to receptor, a conserved tyrosine residue at position 705 (Tyr-705), and a C-terminus encoding the transcription activation domain [8,9]. STAT3 is activated by receptor tyrosine kinases EGFR, HER2, fibroblast growth factor receptor (FGFR), IGFR, HGFR and platelet-derived growth factor receptor (PDGFR), receptor-associated kinases (JAK) and non-receptor kinases (Src and Abl) through phosphorylation [10,11]. While Tyr-705 phosphorylation is critical for STAT3 function, serine 727 (Ser-727) phosphorylation can also occur [12] and has both stimulating and inhibitory effects on gene transcription [13,14,15,16,17]. In addition, Ser-727 phosphorylation may inhibit Tyr-705 phosphorylation [17]. Tyrosine phosphatases in the cytoplasm dephosphorylate STAT3 at Tyr-705 to deactivate its function [18]. STAT3 signaling can also be negatively regulated through two additional pathways. Suppressor of cytokine signaling (SOCS) family inhibits STAT3 at the transcriptional level [19,20]. In contrast, protein inhibitor of activated STAT1 (PIAS1) inhibits STAT3 through direct interaction [21]. Interestingly, although phosphorylation of STAT3 is important for its function, the translocation of STAT3 between the cytoplasm and the nucleus may be independent of the STAT3 phosphorylation status, because of constitutive binding of STAT3 to importin α-3 [22].

## 2. STAT3 in Normal Cells and Development

In normal cells, STAT3 activation is tightly regulated and transient.

### 2.1. STAT3 in Proliferation and Apoptosis

Cell proliferation is the increase in cell number resulting from cell growth and division. Proliferation is induced by growth factors and cytokines, for which STAT3 is an important signaling mediator, as seen with *in vivo* growth hormone treatment’s rapid induction of STAT3 activation via tyrosine phosphorylation [23,24]. Activated STAT3 conveys messages from receptors to the nucleus to modulate the expression of genes involved in cell division. In the neurons of retina, STAT3 couples extrinsic signals with retina precursor cell proliferation [25]. In heart, STAT3 promotes proangiogenic vascular endothelial growth factor (VEGF) expression and growth of myocardial capillaries [26]. Apoptosis, the process of programmed cell death, plays a critical role in development and carcinogenesis. STAT3 positively regulates cell survival by inducing Bcl-2 and Bcl-XL to repress apoptosis [27], and inversely, STAT3 degradation and inhibition cause increased apoptosis [28,29]. IL-6/gp130-mediated cell survival and G1 to S cell-cycle-transition are mediated by the JAK/STAT signaling pathway, and two the STAT3 target genes, *c-myc* and *pim*, are essential for cell survival and cell cycle transition [30].

### 2.2. STAT3 and Differentiation

Differentiation is the process through which cells become more specialized. During tissue repair and normal cell turnover, stem cells divide and differentiate into daughter cells. There is direct evidence that STAT3 induces myogenic differentiation through interaction with MyoD, which is the essential transcription factor in myogenic differentiation [31]. Intriguingly, STAT3 also actively prevents myoblasts from differentiating prematurely, indicating that STAT3 lays an essential role in normal myoblast differentiation through interaction with MyoD and MEF2 [32]. In the neural system, activation of STAT3 by IL-11 induces the expression of glial fibrillary acidic protein, which is a marker for astrocyte differentiation [33]. In addition, STAT3 is essential for motor neuron differentiation in the developing spinal cord [34]. STAT3 also has a central role in the differentiation of osteoblasts and osteoclasts, with overactivation of STAT3 inducing osteosclerosis and suppression of STAT3 leading to osteoporosis [35]. Activation of STAT3 also leads to increased expression of keratin 13, a gene with a critical role in the differentiation of normal human stratified squamous epithelium [36]. Thus, STAT3 is a major regulator of the balance between cellular proliferation and apoptosis.

### 2.3. STAT3 and Immune System

STAT proteins were first discovered in studies of interferons in 1990s and were originally characterized as critical mediators in the immune system [37]. The signaling factors, gp130 [38], IL-6 [39] and leukemia inhibitory factor [40] induce STAT3 tyrosine-phosphorylation and activation, leading to the differentiation of myeloid cells through granulocyte colony-stimulating factor [41,42]. STAT3 also has an essential role in regulating differentiation of B cells into antibody-forming plasma cells [43]. Furthermore, STAT3 activation by IL-6 is required for activation and maturation of dendritic cells (DCs), which are involved in self-antigen tolerance and antigen presentation [44].

### 2.4. STAT3 and Stem Cells

STAT3 is also involved in maintenance and differentiation of stem cells, which because of their capability of self-renewal and differentiation are an important source for tissue repair. There is evidence that STAT3 is one of the signaling components for pluripotent embryonic stem cell-mediated cardiomyogenesis: inhibition of STAT3 drastically suppresses leukemia inhibitory factor-mediated differentiation [45]. STAT3 is also important in maintaining the pluripotential status of proliferating embryonic stem cells.

## 3. STAT3 in Cancer Cells and Cancer Progression

### 3.1. STAT3 in Cancer Cell Proliferation and Apoptosis

Accumulating evidence shows that persistent STAT3 activation is required for aberrant cell proliferation in carcinogenesis. In gastric cancer, IL-26 activated STAT3 signaling induces up-regulated expression of Bcl-2, Bcl-XL and c-Myc, which leads to persistent cell proliferation [46]. The activated STAT3 pathway is involved in cell proliferation of endometrial [47], bladder [48], colon [49] and renal [50] cancers. STAT3 promotes cell survival in esophageal [51], colon [52], gastric [46] and other types of cancer. Unsurprisingly, inhibition of STAT3 results in decreased proliferation and increased apoptosis in cancer cells. Expression of miRNA130b, which targets STAT3, inhibits pancreatic cancer cell proliferation [53]. Inhibitors of STAT3 also suppress cell proliferation and promote apoptosis in breast cancer [54,55], colorectal cancer [56,57], gastric cancer [58] and lung cancer [59]. Based on these studies, STAT3 is a pivotal regulator of cancer cell proliferation and apoptosis.

### 3.2. STAT3 in Angiogenesis

Without new blood vessel formation, the tumor cannot grow beyond the size of 2–3 mm in diameter [60,61]. STAT3 interacts with several factors affecting angiogenesis, a critical step in carcinogenesis that includes degradation of the vascular basement membrane, vascular epithelial cell proliferation and migration, and new vessel reformation and resolution [62]. Inhibition of STAT3 causes decreased angiogenesis mainly through down-regulation of metalloproteinase 2 (MMP-2), which plays an important role in degradation of vascular basement membrane and of basic fibroblast growth factor (bFGF2) and VEGF A, which are critical in vascular endothelial cell proliferation. Conversely, STAT3 activation leads to elevated expression of MMP-2 [63]; it also up-regulates VEGF expression [64,65], since STAT3 binds to the promoter of VEGF directly [65,66,67]. Thus, inhibition of neo-angiogenesis by suppressing STAT3 may represent an attractive strategy in preventing or delaying new tumor formation.

### 3.3. STAT3 and Immune Evasion

Immunosurveillance plays a pivotal role preventing carcinogenesis by detecting and eliminating abnormally transformed cells. However, abnormal cells can evade immune monitoring and eventually form malignant cancers. The many mechanisms by which cancer cell evade detection include reduced expression of cancer antigens and major histocompatibility complex (MHC)-I and MHC-II molecules for T cells and increased immunosuppressive cytokines. STAT3 activation inhibits expression of inflammation cytokines and interrupts maturation of DCs, both processes lead to reduced expression of MHC-II and consequently decreased antigen presentation and T-cell activation [68]. Another line of evidence suggests that STAT3 negatively regulates CXCL10 expression, whose expression significantly enhances natural killer cell cytotoxicity for cancer cells [69]. STAT3 also promotes pro-oncogenic inflammation through nuclear factor-kappa B (NF-κB) and IL-6/GP130/JAK pathways and by inhibiting T helper 1 antitumor immune responses [70]. The involvement of STAT3 in regulating immune evasion may provide an opportunity to prevent the colonization of transformed cells.

### 3.4. STAT3 and Cancer Stem Cells

Cancer stem cells (CSCs), also called tumor initiating cells (TICs), a group of special cancer cells present in tumors, possess characteristics associated with normal stem cells, such as self-renewal and specifically, the ability to generate diverse tumor cells, contributing to tumor heterogeneity. CSCs are responsible for the relapse and metastasis and the resistance to treatment. The function of activated STAT3 in CSCs is suggested by the co-expression of STAT3 with the markers of pluripotent stem cells, Oct 3/4 and Nanog [71]. STAT3 maintains the CSC population and their “stem-like” properties [72]. Selective inhibition of STAT3 by chemical compounds or by siRNA suppresses CSC proliferation [73]. STAT3 may play a role in protecting CSCs from innate immunity as well, since inhibition of phagocytosis and secretion of IL-10 can be reversed upon STAT3 inhibition [74]. Moreover, activated STAT3 is highly correlated with decreased apoptosis and chemoresistance in CSCs [75], and inversely, inhibition of STAT3 results in increased apoptosis and chemosensitivity in CSCs [76,77]. Due to the critical role of STAT3 in maintaining stem cell self-renewal in carcinogenesis, it is rational to speculate that STAT3 blockade may be able to significantly or permanently eliminate CSCs to achieve cancer prevention purpose.

## 4. STAT3 in Malignant Transformation

Malignant transformation—the transition from a normal cell to a cancerous cell—may occur as a primary process in normal tissue or secondarily as malignant degeneration of a benign tumor. Transformed cells have abnormal characteristics compared with the characteristics of their untransformed counterparts. First, after transformation, cells no longer require contact with the surface of a culture dish to grow and a reduction in contact inhibition. Second, transformed cells can grow in multiple layers, whereas normal cells usually grow in a single layer. Third, transformed cells have a decreased requirement for nutrients in media, even in the absence of growth factors. Finally, transformed cells may replicate limitlessly and become immortal. STAT3 is an essential factor in oncogenic cellular transformation, since it is frequently and persistently activated in various types of cancers [8] and constitutive activation of STAT3 is sufficient to convert normal cells into cancer cells [78]. The following three signaling pathways utilize STAT3 activation to transform normal cells into cancerous cells.

### 4.1. G-Protein Coupled Receptor Signaling Pathway

G-protein coupled receptors (GPCRs) can play a role in malignant transformation by inducing STAT3 phosphorylation. After a ligand binds to a GPCR, the GPCR undergoes conformational change and is activated. It then activates an associated G-protein by exchanging the G-protein’s bound GDP for a GTP. The G-protein’s α subunit with the bound GTP then dissociates from the β and γ subunits to affect intracellular signaling proteins or target functional proteins directly. GPCRs may signal through G-protein-independent mechanisms; for example, they may signal independently through Src (see below). Activated by hormones, GPCRs induce phosphorylation of STAT3 [79] and result in increased transformation. Additionally, constitutively activated G-proteins can result in same consequence [80,81]. G-proteins may play functional roles in cell transformation independent of GPCRs. Thus, over-expression of activated G protein α subunits, such as Gao, ai2, aq, a12, and a13, can transform normal cells into neoplastic cells [82,83,84]. The G protein subunits can also activate the STAT3 pathway through Src [85,86]. Src is a cytoplasmic tyrosine kinase containing a catalytic domain, a regulatory domain, and SH2 and SH3 binding domains [87]. The phosphorylated Src at tyrosine 531 stays inactive by masking its own catalytic domain [88]. Dephosphorylation of tyrosine 531 can activate Src, which can activate STAT3 to induce transformation [89,90]. In this pathway, the downstream effector of STAT3 for transformation is c-Myc [91]. Small G proteins with GTPase activity, such as Ras and Ral, can also increase STAT3 phosphorylation as well. Ras induces serine phosphorylation of STAT3 by using the Ras/Raf/MEK-dependent pathway [92]. In contrast, EGF-Ral signaling leads to tyrosine phosphorylation of STAT3 via Src [93].

### 4.2. Receptor Tyrosine Kinase (RTK) Signaling Pathway

Many growth factor receptors, such as EGFR and PDGFR, have an intrinsic kinase domain. Upon binding a ligand, the growth factor receptors become activated and self-phosphorylate tyrosine residues in its cytoplasmic tail [94,95]. Such receptor tyrosine kinases can phosphorylate STAT3 by themselves [96], or they can execute their functions through the JAK/STAT pathway [97,98]. The binding of EGF to EGFR promotes phosphorylation of JAK on tyrosine residues [99] via the intrinsic kinase activity of the EGFR [100]. Additionally, PDGF induces STAT3 phosphorylation via Src protein, and activated STAT3 leads to cell transformation through elevated expression of Myc [91].

### 4.3. Cytokine/JAK/STAT3 Signaling Pathway

IL-6/JAK/STAT3 is the canonical STAT3 activation signaling pathway. Since the receptor of IL-6 does not contain a kinase catalytic domain, it induces STAT3 phosphorylation by activating members of the JAK family [101,102]. Cytoplasmic JAK proteins JAK1, -2, and -3 and Tyk2 are recruited to the tyrosine protein kinases [103,104], which are recruited to the cytoplasmic domain of a ligand-activated receptor, where they phosphorylate the receptor’s tyrosine residues. STAT3 then binds to the phosphorylated receptor-JAK complex via its SH2 domain and is phosphorylated by JAK on a tyrosine residue near the C terminus [98,105]. IL-6/JAK/STAT3 signaling can induce malignant transformation via STAT3 activated up-regulation of miR-21 in an autocrine manner [106].

## 5. STAT3 Contributes to Early Carcinogenesis in Animal Models

The findings of several *in vivo* studies also indicate that STAT3 contributes to tumorigenic transformation. One study used a K5.Cre × STAT3^flx/flx^ mouse model of skin-specific STAT3 deficiency to show that loss of STAT3 led to significant reduction of epidermal hyperproliferation due to the down-regulated expression of *c-myc* [107]. Moreover, K5.STAT3C transgenic mice expressing constructively active STAT3, form tumor faster and a much greater number than do their nontransgenic counterpart. Remarkably, all of the skin tumors from K5.STAT3C transgenic mice bypassed the premalignant stage, implying relatively easy transformation [108]. Another transgenic mice model, K5.CreER^T2^, with a tamoxifen inducible Cre gene under the control of K5 promoter, was used to study STAT3 effect on tumor transformation in a temporally controlled and inducible pattern. The study demonstrated that temporal disruption of STAT3 at initiation stage of skin carcinogenesis could promote apoptosis and prevent transformation by inhibiting Bcl-XL [109]. Similarly, tumorigenesis is much less in mice with heterozygous STAT3 gene (STAT3 (±): HPV8) than in mice with wild-type epidermis. Furthermore, the tumors in the STAT3 (±): HPV8 mice are benign and never transformed to a more malignant phenotype [110]. Finally, ARR_2_Pb.STAT3C mice, expressing constructively activated STAT3 in prostate, exhibit significant hyperplasia and intraepithelial neoplasia in the prostate. Sixty percent of STAT3+/Pten+/− mice developed prostate tumors within 12 months of age [111]. These studies suggest that STAT3 is a positive contributor in the early phases of carcinogenesis, and thus provide *in vivo* evidence that STAT3 could serve as a target for preventive intervention.

## 6. Role of STAT3 in Cancer Prevention

Although STAT3 is constitutively active in numerous types of cancers including breast cancer, skin cancer, ovarian cancer, prostate cancer, multiple myeloma, lymphomas and leukemia, brain tumor, Ewing sarcoma, gastric cancer, esophageal cancer, colon cancer and pancreatic cancer, one report showing STAT3 mutation in the SH2 domain in large granular lymphocytic leukemia, resulting in an increased activity of the protein [112]. No other mutation in STAT3 was reported. STAT3 can be maintained in a constitutively active status only through deregulation of signaling molecules by the proteins that negatively regulate STAT3. SOCS proteins inhibit STAT3 at the transcriptional level [19,20]. In contrast, PIAS1 inhibits STAT3 through direct interaction [21]. In addition, STAT factors modulate their own protein expression and cross-regulate among themselves. STAT3 may also regulate expression of STAT1, STAT3, STAT5a, STAT5b, STAT6, and upstream kinases JAK2 and JAK3, through single or multiple cis-element(s) in their promoters. Thus, STAT3 is a major regulator for the STAT/JAK signaling pathway and the convergent point of transforming signaling during carcinogenesis.

### 6.1. STAT3 Regulates Stem Cell-Like Breast Cancer Cells

Accumulating evidence indicates that STAT3 plays a key role in maintenance of stem cell-like breast cancer cells, which have been shown to be related to tumor recurrence, metastasis and chemo-resistance [75,113]. Different markers were used to show that the CD44+/CD24− phenotype is more prevalent for breast stem-like cells [114]. Lauren *et al.* found that gene expression pattern differs between CD44+/CD24− and CD44−/CD24+ populations. The IL-6/JAK2/STAT3 pathway was found to be active in CD44+/CD24− breast cancer cells, and inhibitors blocking this pathway suppressed growth of xenograft tumors. In addition, it has been reported that a non-CSC population can be converted into a CSC-like population via the IL-6/JAK1/STAT3 signaling pathway through upregulating of OCT-4 [115]. STAT3 inhibitors, such as niclosamide, were found to prevent the production of OCT-4. Therefore, these studies support that STAT3 plays a pivotal role in regulating the self-renewal of stem cell-like breast cancer cells.

### 6.2. Targeting STAT3 for the Prevention of ER-Negative and Triple-Negative Breast Cancer

Several studies suggest that STAT3 represents a promising molecular target for the prevention of ER-negative and triple-negative breast cancers (TNBC). STAT3 plays a critical role in the development of estrogen receptor (ER)-negative breast cancer. ER, a member of the nuclear receptor superfamily, is a ligand-activated transcriptional factor [116] and plays an important role in the differentiation and development of various organs. Thus, it is not surprising that ER is critical in the transformation of normal breast epithelium to malignant and invasive breast cancer cells. There is evidence that ER has a ligand-independent function, which can prevent cells from apoptosis induced by serum deprivation by activating the c-Src/STAT3 signaling pathway [117]. In turn, STAT3 can enhance the transactivation of ER [118]. A significant correlation between STAT3 Ser-727 phosphorylation and ER-negative breast cancer has been identified. The levels of phospho-Ser-727-containing STAT3 are significantly higher in ER-negative breast cancer cell lines than ER-positive breast cancer cell lines [119]. One possible explanation is that ER, through direct protein-protein interaction with STAT [120], can inhibit STAT3 Ser-727 phosphorylation and thus suppress STAT3 activity. Moreover, estrogen-activated ERα can up-regulate the transcription of PIAS3, which directly binds to STAT3 and inhibits its function [121].

On the other hand, acetylation at the STAT3 K685 site enables STAT3 interaction with DNA methyltransferase 1 (DNMT1) and directs DNMT1 to the ERα gene promoter; then DNMT1 methylates the CpG islands in the promoter of ERα gene and suppresses its transcription [122]. These confounding findings need to be explored in detail to determine the role of STAT3 in carcinogenesis of TNBC, which is defined by the lack of ER, PR and low expression of HER2 which represents about 15%–20% of breast cancers [123]. In addition, phosphorylation of Ser-727 in STAT3 is required for transactivation by association with p300/CREB-binding protein [124]. This transactivation increases STAT3 transcriptional activity and thus promotes breast cancer carcinogenesis. These studies suggest a critical role of STAT3 in the development of ER-negative breast cancer and TNBC, therefore, STAT3 represents a promising molecular target for the prevention of these breast cancer subtypes.

### 6.3. Targeting STAT3 for the Prevention of ER-Positive, SERM/Aromatase Inhibitor-Resistant Breast Cancer

Selective estrogen receptor modulators (SERMs) are ER ligands that have selective functions in different tissues. For example, in bone and cardiovascular system, they act like estrogen, whereas in others tissues, they block estrogen action. The classic SERMs tamoxifen and raloxifene have both been shown to help prevent ER-positive breast cancer [125]. Because SERMs achieve their preventive effect through binding to ERα, resistance to SERMs may result from loss of ER expression. Competitive binding to ER is another important mechanism of SERM resistance, especially in TNBC. There is evidence that cyclin D1 binds and activates ER in a ligand-independent manner [126,127]. Thus overexpression of cyclin D1 may cause resistance to tamoxifen [128,129]. As a positive regulator of cyclin D1, STAT3 therefore plays an important role in resistance to SERMs [130]. Activated STAT3 is translocated to the nucleus, where it induces transcription of AP-1. In turn, AP-1 induces transcription of aromatase, which turns androgens into estrogens in adipose tissue, and estrogen activates its membrane receptor ERα and stimulates the proliferation of breast epithelial cells [130]. Phosphorylated STAT3 binds to the promoter of aromatase-encoding CYP19 gene and stimulates the expression of P450 gene; consequently, estrogen biosynthesis increases in human adipose tissue [130]. Thus, blocking STAT3 might be an effective route to prevent ER-positive breast cancer, via several potential mechanisms different from those used by SERMs or aromatase inhibitors. This strategy may be particularly useful against ER-positive breast cancers that do not respond to SERMs.

### 6.4. Targeting STAT3 for the Prevention of Other Cancer Types

In addition to breast cancer, STAT3 stays constitutively activated in other types of cancers. STAT3 has been shown to be constitutively activated or overexpressed in colon cancers [131]. STAT3 is activated in nearly 50% of lung cancer [132]. IL-6 and STAT3 overexpression were seen in prostate cancer and blockade of STAT3 suppressed clonogeneity in stem cell-like cells in high grade prostate cancer patients [133]. SiRNA against STAT3 eradicated established AML and impaired the potential of leukemia-initiating cells; further, LLL12 treatment abolished outgrowth of a patient-derived castrate-resistant tumor [134]. Taken together, STAT3 is required and essential in tumorigenesis of a variety of cancers. We therefore predict that blocking STAT3 signaling with STAT3 inhibitors, perhaps in combination with other inhibitors targeting STAT3 upstream signal molecules, may serve as a general preventive strategy for the prevention of individual or multiple cancer type(s). Thus, STAT3 inhibitors may be used as chemopreventive agents for the prevention of cancers of colon, lung, prostate, pancreas, ovary and hematopoietic origin.

## 7. STAT3 Inhibitors as Potential Cancer Preventive Agents

As mentioned earlier, STAT3 regulates the expression of various genes involved in proliferation, apoptosis, angiogenesis, invasion, and metastasis [135]. Skin carcinogenesis studies with STAT3-deficient mice showed that STAT3 activation is required for both the initiation and promotion stages [107]. Therefore, inhibition of STAT3 provides a rational strategy to block carcinogenesis at early stage of cancer development. Development of STAT3 inhibitors is currently demanded for either cancer prevention or treatment. A major focus of this review is the potential cancer prevention role offered by STAT3 inhibitors or agents, although there are no FDA-approved STAT3 inhibitors available for clinical use to date. Here, we summarized the currently available general and/or specific STAT3 inhibitors in Table 1 for quick reference. Two major strategies are used to inhibit the STAT3 signaling pathway: (**1**) Direct inhibition of STAT3 protein with inhibitors targeting one of three structural domains of STAT3, namely (**a**) SH2 domain, (**b**) DNA binding domain, and (**c**) N-terminal domain, which suppresses processes related to STAT3 signaling and functional role by blocking phosphorylation, dimerization, nuclear translocation and DNA binding [136,137]; or (**2**) Indirect blocking the upstream regulators of STAT3 pathway.

### 7.1. SH2 Domain Inhibitors

The SH2 domain of STAT3 plays an essential role in STAT3 activation by mediating the interaction of STAT3 with phosphorylated tyrosine residues on the cytoplasmic portion of receptors. The SH2 domain also plays a critical role in the formation of STAT3 dimer, where the SH2 domain of one STAT3 monomer binds to the phosphorylated tyrosine (pTyr) motif of the other. Thus, inhibition of SH2 domain suppresses the phosphorylation and activation of STAT3 protein. Peptidomimetics, a major category of inhibitors that binds to the SH2 domain of STAT3, mimic pTyr-Xaa-Yaa-Gln motif and inhibit STAT3 dimerization by competitive binding to the SH2 domain [138]. The phosphopeptide inhibitor is derived from the STAT3 SH2 domain-binding peptide sequence, PY*LKTK (Y* is the phosphorylated tyrosine). This small-molecule peptide can directly form a complex with STAT3 monomer and inhibit STAT3 activity by disrupting STAT3 dimerization and consequently.

**Table 1:** Currently Available STAT3 Inhibitors.

Inhibitor Name	Mechanism of Action	Selectivity	Cancer Type	Reference
PY*LKTK	SH2 domain inhibitor	STAT3	NIH 3T3/v-Src fibroblasts	[139]
S31-M2001	SH2 domain inhibitor	STAT3	Breast cancer	[140]
S31-1757	SH2 domain inhibitor	STAT3	Breast and lung cancer	[141]
Curcumin-proline	SH2 domain inhibitor	STAT3		[142]
Cryptotashinone	SH2 domain inhibitor	STAT3	Prostate cancer	[143]
STA-21	SH2 domain inhibitor		Breast cancer	[144]
Stattic	SH2 domain inhibitor	STAT3	Breast cancer	[145]
S3I-201	SH2 domain inhibitor	STAT3	Breast cancer, prostate cancer, acute myeloid leukemia and human multiple myeloma	[146]
BP-1-102	SH2 domain inhibitor	STAT3	Breast and Lung cancer	[147]
Celecoxib	SH2 domain inhibitor	STAT3	Human rhabdomyosarcoma	[148]
SPI	SH2 domain inhibitor	STAT3	Breast, pancreatic, prostate, and non-small cell lung cancer cells	[149]
HIC 1	DNA binding domain inhibitor	STAT3	Breast cancer	[150]
IS3-295	DNA binding domain inhibitor	STAT3	Colon tumor	[151]
DBD-1	DNA binding domain inhibitor	STAT3	Melanoma	[152]
ST3-H2A2	N-terminal domain inhibitor	STAT3	Prostate cancer	[153]
G-quartet ODN	Oligonucleotide inhibitor	STAT3	Head and neck, breast and prostate cancers	[154,155,156,157]
SiRNA to STAT3	SiRNA	STAT3	Laryngeal, breast, lymphoma, prostate and melanoma	[158,159,160,161,162]
KDI1	RTK inhibitor	STAT3	Vulval and breast cancer	[163]
PD153035	RTK inhibitor	STAT3	Oral squamous carcinoma	[164]
Ponatinib	FGFR inhibitor	STAT3	Rhabdomyosarcoma	[165]
AG490	JAK kinase inhibitor	STAT3	Pancreatic cancer	[166]
SHP1	STAT3 inhibitor	STAT3	Multiple myeloma and head and neck squamous carcinoma cells	[167]
WP1066	JAK kinase inhibitor	STAT3	Acute myelogenous leukemia	[168,169]
TG101209	JAK2 kinase inhibitor	STAT3,5	Acute myeloid leukemia	[170]
AZD1480	JAK kinase inhibitor	STAT3	Myeloma,Neuroblastoma and Pediatric Sarcomas	[171,172]
Dasatinib	Src and PDGF inhibitor	STAT3	Synovial sarcoma, hepatocellular carcinoma, glioma, prostate cancer	[173,174,175,176,177]
PP2	Src inhibitor	STAT3 and Src	Intestinal epithelial cell	[178]
KX2-391	Src inhibitor		Prostate cancer	[179]
AZD0530	Src inhibitor	STAT3	Melanoma	[180]
E738	Src and JAK inhibitor	STAT3	Pancreatic cancer	[181]
MLS-2384	Src and JAK inhibitor	STAT3	Prostate, breast, skin, ovarian, lung, and liver cancer	[182]
Sophoraflavanone G	Src and JAK inhibitor	STAT3,5	Breast, prostate, lymphoma,human multiple myeloma,large cell lung cancer, colorectal carcinoma	[183]
SHP2	STAT3 inhibitor	STAT3	Chronic myeloid leukemia	[184]
HJC0152	STAT3 inhibitor	STAT3	Breast cancer	[54,55]
HJC0123	STAT3 inhibitor	STAT3	Breast cancer	[54,55]
Xanthohumol	STAT3 and EGFR inhibitor	STAT3	Breast cancer	[185]
Brevilin A	JAKs inhibitor	STAT3	Breast cancer	[186]
Benzyl isothiocyanate	STAT3 inhibitor	STAT3	Breast and pancreatic cancer	[187]

STAT3-dependent transcription in both human and mouse cells [139]. A novel oxazole-based peptidomimetic, S3I-M2001, selectively disrupts STAT3 dimerization and consequently inhibits STAT3 dependent transcription, transformation, survival and migration in both human and mouse cells [140]. Another peptidomimetic, S3I-1757, a derivative of benzoic acid, blocks hyperactivated STAT3 and suppresses malignant transformation by disrupting STAT3-STAT3 dimerization via direct interaction with Tyr-705 binding site in the STAT3-SH2 domain [141].

Another group of SH2 domain blockers is derived from the natural compound curcumin. Modification of curcumin with proline produces the most potent conjugate inhibitor of STAT3 dimerization [142]. Cryptotanshinone is also a natural compound with the ability to bind to SH2 domain and inhibit formation of STAT3 dimers. The binding of cryptotanshinone to SH2 domain of STAT3 inhibits STAT3 phosphorylation and, in turn, decreases the expression of STAT3 downstream target genes, such as cyclin D1, survivin, and Bcl-XL, involved in cell survival [143].

Because phosphopeptides have low cell penetration efficiency, investigators have explored the potential of a new series of small molecules: STA-21, stattic, S3I-201 and BP-1-102, as STAT3 SH2 domain inhibitors. STA-21 is a natural deoxytetrangomycin, an angucycline antibiotic, that specifically binds to SH2 domain and abrogates STAT3 dimerization and nuclear translocation, thereby significantly inhibits breast cancer cell growth and survival [144]. Stattic, a nonpeptidic small molecule, selectively inhibit dimerization and DNA binding of STAT3, via preventing activating enzymes to the STAT3 SH2 domain. As a result, stattic induces apoptosis in breast cancer cell lines [145]. S3I-201, a low-molecular-weight salicylic acid derivative, disrupts STAT3-STAT3 interaction through SH2 domain binding [146] and induces apoptosis in malignant cells by suppressing STAT3-dependent expression of cyclin D1, Bcl-XL, and survivin [188]. BP-1-102, an analog of S3I-201, inhibits STAT3 through the same mechanism by binding to the SH2 domain and selectively suppresses malignant cell growth, survival, transformation, migration, and invasion in human breast and lung cancer cells [147].

Interestingly, celecoxib, a cyclooxygenase-2 inhibitor, also binds to the SH2 domain of STAT3 and competitively inhibit native peptide binding, leading to suppressed tyrosine phosphorylation and reduced cell viability and migration in human rhabdomyosarcoma cells [148]. Another class of STAT3 dimerization inhibitors is derived from salicylic acid. The advantage of this class STAT3 inhibitor is a higher cell membrane permeability than that of peptidomimetics. Salicylic acid derivatives effectively disrupt STAT3-phosphopeptide complexes and inhibit STAT3-STAT3 interactions. As a result, these prevent intracellular STAT3 phosphorylation and promote cell death in human cancer cell lines [189]. Another SH2 domain blocker, SPI, which is a 28-mer peptide derived from the STAT3 SH2 domain. potently and selectively inhibits STAT3 SH2 domain interaction with the pTyr residue on the cytoplasmic tail of IL-6R, thereby inhibiting STAT3 tyrosine phosphorylation and inducing apoptosis in multiple cancer cell lines [149].

Phosphorylation of Tyr-705 in the SH2 domain is not only required for dimerization, but also important in STAT3-DNA binding. Therefore, STAT3 inhibitors that target SH2 domain also inhibit STAT3-DNA interaction. For example, in targeting the SH2 domain, S3I-1757 interferes with STAT3-DNA binding, and this interference suppresses the expression of STAT3 target genes, including Bcl-XL, survivin, cyclin D1, and MMP-9, thereby promoting apoptosis and inhibiting proliferation [141]. This effect can be used to block carcinogenesis at early stage of transformation. Like S31-1757, S3I-201 and its analogues also potently inhibit STAT3-DNA binding through interaction with the SH2 domain [146].

### 7.2. Inhibitors for STAT3 DNA Binding Domain

To regulate gene expression, it is essential for STAT3 to bind to the gene’s promoter. Thus, STAT3 activity can potentially be inhibited by targeting the STAT3 DNA binding domain to prevent interaction with the target gene’s promoter. While this strategy is plausible in theory, it has not been adequately explored, possibly because targeting the SH2 domain can induce multiple inhibitory effects on STAT3 function. Endogenously, the HIC1 (Hypermethylated in cancer 1) gene, a tumor suppressor, forms complex with STAT3 protein through direct interaction between the C-terminal domain of HIC1 and the DNA binding domain of STAT3. The net result of this interaction is prevention of STAT3 binding to the promoter of the target genes, such as the genes encoding VEGF and c-Myc [150].

One new platinum (IV) compound, IS3-295, inhibits the DNA-binding capability of STAT3 in a non-competitive manner; the mechanism it uses remains unclear. Other platinum (IV) compounds, such as CPA-1, CPA-7 and platinum (IV) tetrachloride, also inhibit STAT3 DNA-binding, thereby suppressing cell growth and increasing apoptosis in several types of human cancer cells [151]. Further investigation is needed to determine whether these platinum derivatives directly bind to the STAT3 DNA-binding domain to execute their function.

DBD-1, a small peptide aptamer, can specifically recognize the DNA binding domain in STAT3 *in vitro*. Although *in vivo* findings showed only weak interaction between DBD-1 and STAT3 DNA binding domain, DBD-1 can induce significant apoptosis in murine melanoma B16 cells [152].

### 7.3. Inhibitors Blocking STAT3 N-terminal Domain

The N-terminal domain of STAT3 has eight helices, which have multiple biological activities, including dimer formation, binding to promoter and assembly of transcriptional machinery [190,191]. Compounds targeting the N-terminal domain of STAT3 may therefore inhibit tumorigenesis. One peptide derived from helix-2 of STAT3 can specifically bind to the N-terminal domain of STAT3 and induce cell death in multiple breast cancer cell lines [191]. The mechanism is by apoptosis, since ST3-H2A2, a synthetic compound, binds to STAT3 N-terminal domain and activates expression of proapoptotic genes such as C/EBP-homologous protein (CHOP) to initiate apoptotic death in cancer cells [153].

All the inhibitors discussed above may interact with STAT3 protein directly to execute their inhibitory functions. However, the STAT3 signaling pathway is a multiple-component cascade, other inhibitors may suppress STAT3 protein by indirect mechanisms: decoy oligodeoxynucleotide can block STAT3 binding to its target gene promoters, siRNAs can inhibit STAT3 mRNA translation, and inhibitors of upstream kinases such as JAK and Src can suppress STAT3 phosphorylation and activation.

### 7.4. Oligonucleotide Inhibitors for STAT3

A STAT3-decoy oligonucleotide (ODN) can trap an activated STAT3 dimer in the cytoplasm by inhibiting interaction between active STAT3 and importin, resulting in increased apoptosis in colorectal cancer cells [192]. Another mechanism used by ODN is competitive prevention of STAT3 binding to its targeted gene promoter. Synthetic double-stranded ODN containing consensus sequence of the *cis*-element can bind to transcription factor with high affinity. The decoy ODN competes with the endogenous *cis*-elements for the binding site of the targeted transcription factor, thereby down-regulating target gene transcription by STAT3. This strategy has been proven useful in *in vitro* and *in vivo* studies, with ODNs blocking STAT3-dependent transcription of cyclin D1, c-Myc, Bcl-XL, and survivin [193]. The reduced expression of these gene products inhibits proliferation and increases apoptosis in a variety of tumor cell lines [194,195] and induces tumor regression in xenograft models [196,197]. The G-quartet ODN, which forms four-stranded G-quartet structures [154], uses another mechanism: it disrupts STAT3:STAT3 dimers and inhibits STAT3 binding to DNA [155], thereby inducing tumor cell apoptosis and tumor regression [156,157,193]. Another effective method of blocking STAT3 activity with ODNs is the use of siRNA to degrade STAT3 mRNA. SiRNA targeted at STAT3 mRNA induces increased apoptosis in many types of cancer cells via down-regulation of antiapoptotic proteins, including Bcl-2 and Bcl-XL [158,159,160] and up-regulation of pro-apoptotic proteins, such as Fas, Fas-L, caspase-3 and Bax [159,161], via recruiting FADD, which forms complex with caspase 8, Fas, and Fas-L to activate apoptotic pathways, sequential caspase activation to induce mitochondrial dysfunction, and Bax translocation. In addition, STAT3 siRNA inhibits tumor cell growth and proliferation by down-regulating c-Myc and cyclin D1 [162]. Challenges remain for ODNs in delivering ODNs in cancer prevention settings.

### 7.5. Inhibitors of Receptor Tyrosine Kinase Activity

Peptide aptamer KDI1 forms complex with EGFR through interaction with its intracellular domain. KDI1 does not block EGFR’s RTK activity directly but interferes with EGF-induced phosphorylation of Tyr-705 in STAT3 protein [163]. Another EGFR inhibitor, PD153035, inhibits phosphorylation of EGFR and STAT3 *in vivo* and prevents oral squamous cell carcinoma growth and proliferation [164]. FGFR is also a cell surface tyrosine kinase receptor. Ponatinib inhibits FGFR phosphorylation and subsequent STAT3 phosphorylation, inducing increased apoptosis in rhabdomyosarcoma cells and inhibition of tumor growth in mice [165].

### 7.6. Inhibitors for JAK and Src Kinases

Since phosphorylation is essential for STAT3 biological activity, blocking the upstream kinases responsible for STAT3 phosphorylation represents a rational approach to prevent carcinogenesis. STAT3 is phosphorylated by several protein kinases located in the cytoplasm. Here, we focus on two classical kinases, JAK and Src.

JAK inhibitors AG490 [166,198], WP1066 [168,169], TG101209 [170] and AZD1480 [171,172] reduce the level of STAT3 phosphorylation, resulting in multiple anticancer effects such as decreased tumor growth, increased apoptosis, and reduced metastasis. In addition to inhibit JAK, AG490 suppresses the IL-6/JAK/STAT3 signaling pathway by downregulated translation of gp130, a common receptor component for the IL-6 cytokine family [198].

The Src inhibitor dasatinib also decreases STAT3 phosphorylation [173,174], causing decreased cell growth and proliferation [175], increased apoptosis [176] and inhibited metastasis [177]. Another Src inhibitor, PP2, inhibits phosphorylation of both Src and STAT3 [178]; the resulting accelerated differentiation of intestinal epithelial cells can prevent carcinogenesis [199]. Two promising Src inhibitors, KX2-391 [179] and saracatanib (AZD0530) [180], are currently in phase II clinical trials.

A few newly developed compounds exert their antitumor effects through synergic inhibition of multiple cytoplasmic kinases. The indirubin derivative E738 [181], 6-bromoindirubin derivative MLS-2384 [182] and sophoraflavanone G [183] inhibit both JAK and Src. These inhibitors may therefore efficiently inhibit cancer cell growth and induce apoptosis.

Since tyrosine phosphorylation is essential for STAT3 activity, removal of the phosphate moiety from STAT3 by phosphatase can inhibit STAT3 signaling. SHP1 [167] and SHP2 [184] dephosphorylate phosphorylated-STAT3, resulting in increased apoptosis and decreased angiogenesis [167,184].

### 7.7. Natural Products that Inhibit STAT3 Activity

There are plenty of natural products that can inhibit STAT3 activity, in addition to inhibit other target molecules such as AP-1 and NF-κB. Several natural or dietary compounds have been shown to possess *in vitro* and/or *in vivo* inhibitory effect against STAT3. Curcumin as a non-specific STAT3 inhibitor reduces cell proliferation and colony formation in cell culture and preclinical model in lung cancer via reducing the phosphorylation of STAT3 and inhibit the activation of STAT3 [132]. Curcumin was also reported to be able to reverse inhibition of constitutive STAT3 in multiple melanoma cells and inhibit the translocation of STAT3 in multiple melanoma cells [200]. Xanthohumol is a natural flavonoid compound and non-specific inhibitor for STAT3 and EGFR, it sensitizes MCF-7/ADR cells to apoptosis via upregulating DR5/DR4 and suppresses antiapoptotic proteins [185]. Brevilin A, a pseudoguaiane sesquiterpene from *Litsea glutinosa*, showed significant inhibition of STAT3 activity by inhibiting JAK via blocking tyrosine kinase domain JH1 in AKs [186]. Benzyl isothiocyanate, an extract from cruciferous vegetables, blocks STAT3 activation induced by IL-6 in MDA-MB-453 breast cancer cells and PANC-1 pancreatic cancer cells [187]. Natural products are generally well tolerated, an advantage in cancer prevention setting. However, the non-specific effect on targeting STAT3, off-target effects, variable bioavailability and moderate efficacy constitute disadvantages for natural products as effective chemopreventive agents. Yet new opportunities are still highlighted when structural modification are made in natural products to derive novel chemopreventive drug candidates, while avoiding the aforementioned shortcomings of natural products as STAT3 inhibitors.

### 7.8. Orally Bioavailable STAT3 Inhibitors through Fragment-Based Drug Design Approach

In the past decade, fragment-based drug design (FBDD) has been an efficient strategy for generating novel lead molecules against therapeutic targets [55]. New chemical leads are identified by merging privileged fragments to bind cooperatively in a binding pocket. These fragments keep their original orientations while providing enhanced binding [55]. Niclosamide, an FDA-approved anticestodal drug which has a very low bioavailability in human [55], was recently identified to have significant inhibition on STAT3 activation, nuclear translocation and transactivation [201]. Using FBDD, our team has generated a series of orally bioavailable STAT3 inhibitors such as HJC0152 and HJC0123 based on the structure of niclosamide and others [54,55]. These novel STAT3 inhibitors show significant inhibition of STAT3 activation, STAT3 reporter promoter activity, and the growth of breast and pancreatic cancer cell lines *in vitro* and xenograft breast tumors derived from TNBC MDA-MB-231 cells, via oral administration [54]. In our ongoing study, HJC0152 effectively prevents ER-negative breast tumor formation in a transgenic mouse model.

## 8. Conclusions: Future Direction and Perspective

**Figure 2:**
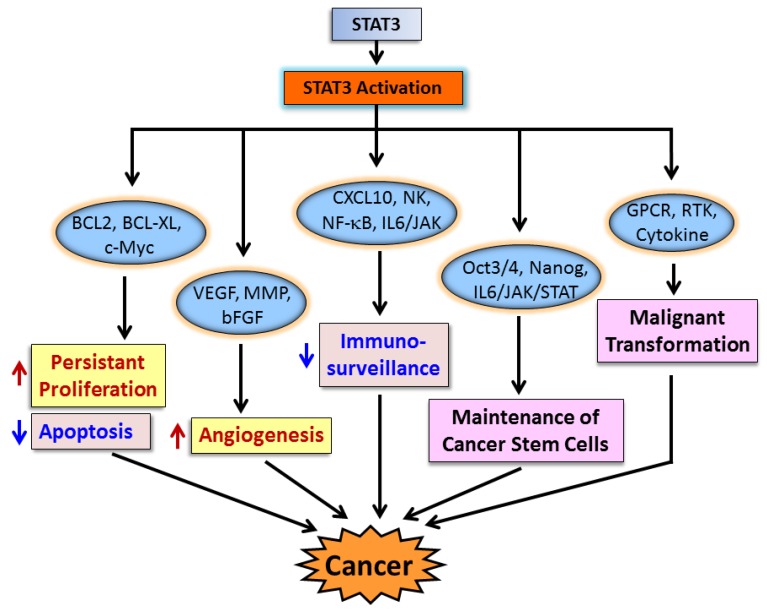
Schematic mechanisms stat3 contributing to carcinogenesis.

Various human cancers, including breast cancer, may be susceptible to the preventive or therapeutic effects of STAT3 inhibitors. Aberrant STAT3 signaling promotes carcinogenesis and tumor progression through deregulation of the expression of downstream genes that control the proliferation, apoptosis, angiogenesis, metastasis, immunesurveillance, malignant transformation and maintenance of CSCs. These genes include the ones encoding (**1**) Bcl2, Bcl-XL and c-Myc, which promote proliferation and cancer cell survival and inhibit tumor cell apoptosis; (**2**) VEGF, MMP, and bFGF, which promote angiogenesis; (**3**) CXCL10, NF-κB and IL6/JAK, which are involved in immunesurveillance; (**4**) Oct3/4, Nanog and IL6/JAK/STAT3, which maintain CSC functions; and (**5**) GPCR, RTK and cytokines, which are responsible for malignant transformation (Figure 2). Targeting activation of STAT3 in premalignant cells therefore represents a promising strategy for preventing human cancers.

The current status of cancer chemoprevention is well summarized in recent literature [202,203,204]. To achieve effective chemoprevention against major cancer types, including breast cancer, lung cancer, colorectal cancer, and prostate cancer, targeted preventive therapies are urgently needed. Transcription factors such as STAT3 represent a group of promising molecular targets for preventive intervention for multiple cancer types. However, neither the use of STAT3 inhibitors as chemopreventive agents nor FDA-approved STAT3 inhibitors have been reported to date. Breast cancer may be an ideal cancer type to test the chemopreventive efficacy of STAT3 inhibitors, given STAT3 involvement in early mammary tumorigenesis of ER-negative and TNBC subtypes. Development of novel STAT3 inhibitors will present enormous opportunities in the cancer prevention field. Potential challenges include long-term effectiveness for populations at high risk of developing various cancers, low toxicity for long- or short-term administration, and ease of use for compliance to prescribed regimens.
